# Supplementary figures and images for: Genetic grouping of SARS-CoV-2 coronavirus sequences using informative subtype markers for pandemic spread visualization
Source: PLoS Comput Biol. 2020 Sep 17;16(9):e1008269. doi: 10.1371/journal.pcbi.1008269 (PMC7523987; doi:10.1371/journal.pcbi.1008269)

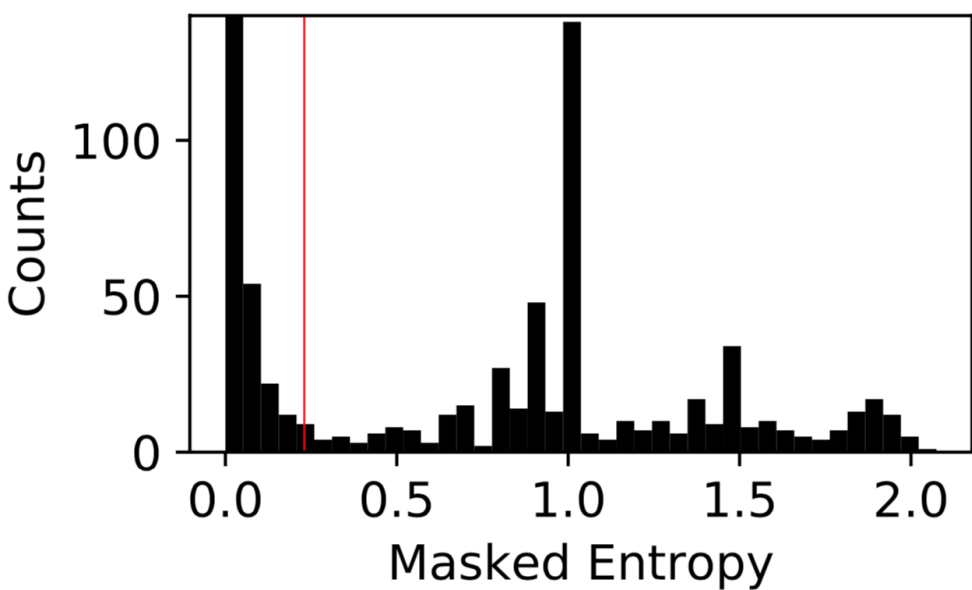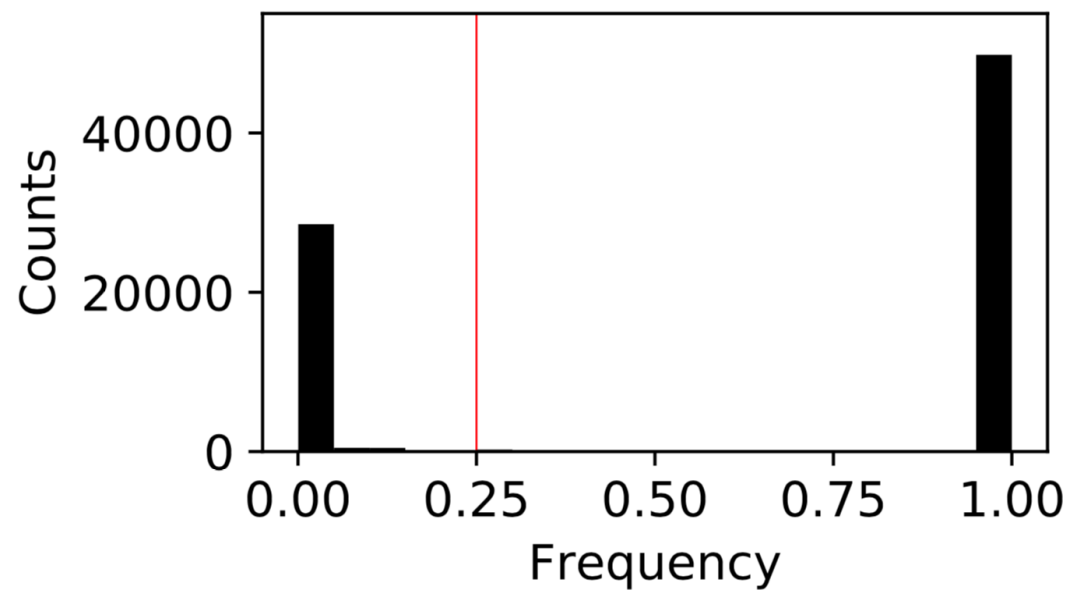

Supplement: S1 Fig — The red line demonstrates the high-entropy threshold used to define ISM sites (> 0.23); Right: Many genome positions in the alignments had nearly all ambiguous bases and/or gaps (the peak at around 1.0). On the other hand, the peak at around 0 represents high quality positions with a fewer number of N and - present. Sites with large number of N’s and - should be filtered out because a large number of N’s and -’s at a position is typically due to sequencing error or alignment artifact which provides less information about the real nucleotide distribution at this position. We set the percentage of N and - threshold to < 0.25 (indicated by the red vertical line in this plot) to keep the most informative group of sites in the genome). The left hand side of the plot shows that there are over 100 positions with an entropy value around 1 (here we show the counts of entropy values greater than 0.1 because most of the positions in viral genomes have no variation so far and thus leads to 0 entropy at those positions—the peak at 0 masked entropy is not shown in full). However, most of those positions have high entropy because there are high percentage of N and - at those positions across all sequences in our dataset. The right hand side of the plot shows that there are a large amount of positions with high frequency of N and - (the peak at around 1.0). On the other hand, the peak at around 0 represents high quality positions with a fewer number of N and - present. According to this figure, we set the percentage of N and - threshold to 0.25 to keep the most informative group of sites in the genome. (PDF) [file pcbi.1008269.s001.pdf]

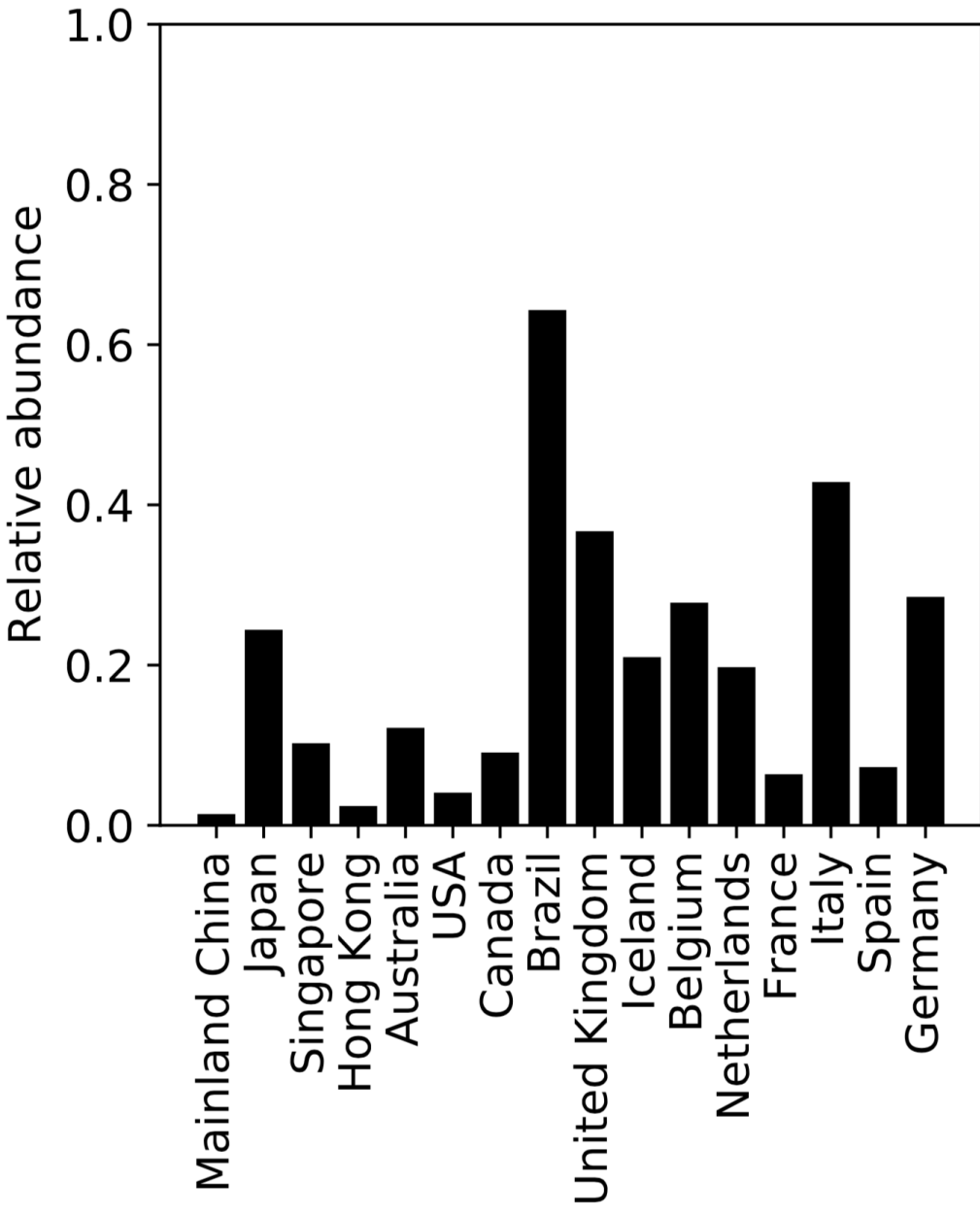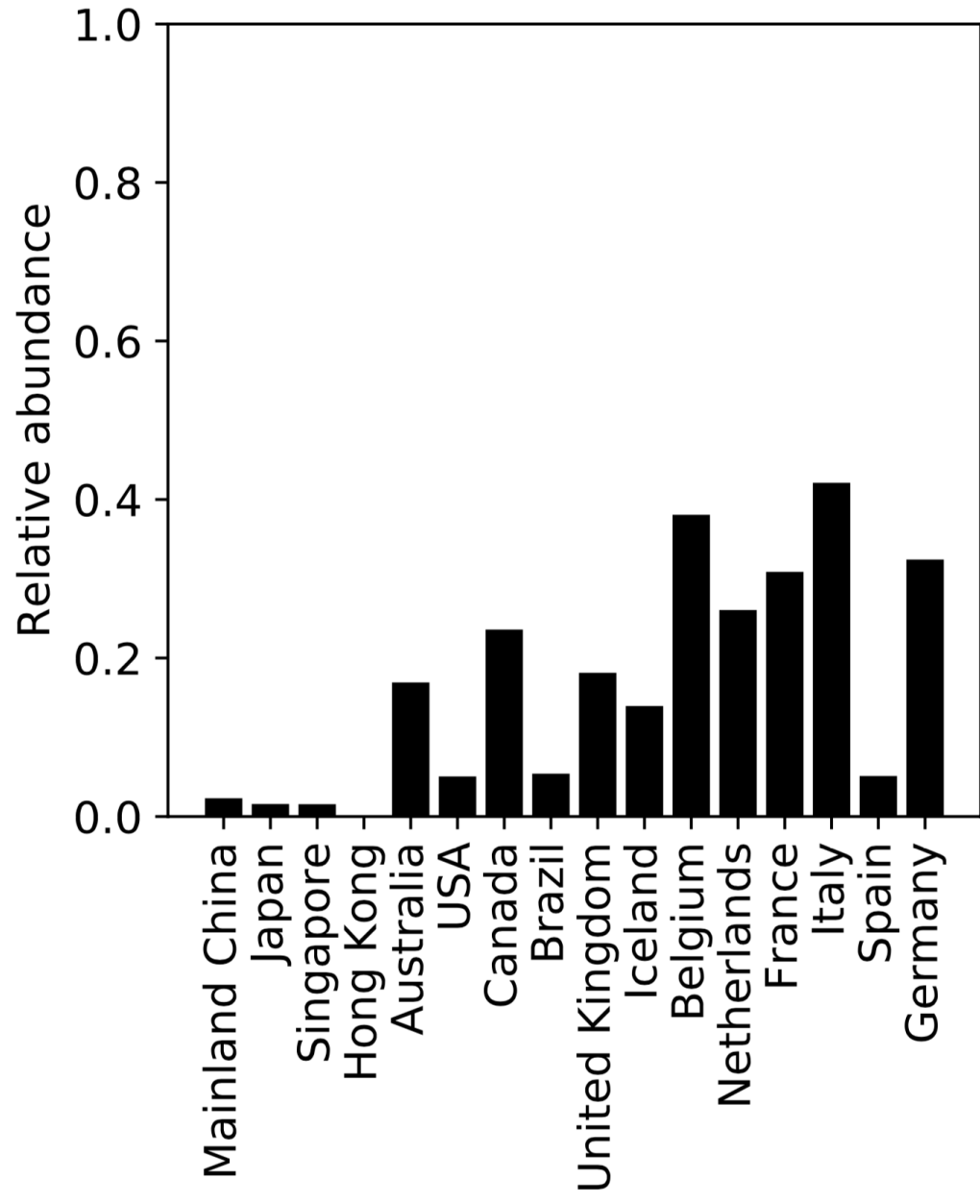

Supplement: S2 Fig — Based on publicly available sequence data from Italy, we found that Italy had two particularly abundant ISMs, CCCGCCAGGGA and CCCGCCAGGGG, as can be seen in the pie chart in Fig 5. The third-most abundant subtype shown in the chart (CCCTCCAAGTG) corresponds to cases that were linked to original exposure from China, which is consistent with the ISM being in common with one found in Hong Kong. This supplementary figure shows the relative abundance (proportion of total sequences in that country/region) of each of these two dominant subtypes of Italy in other countries/regions. As the plot shows, the outbreak in other European countries have generally involved the same viral subtypes as those which are most abundant in Italy, as defined by ISM. Indeed, initial reports of cases in various other European countries in late February 2020 were linked to travelers from Italy [68]. The subtypes which are predominant in Italy are found, however, at lower yet notable abundance in countries including Japan, Canada and Australia. Somewhat surprisingly, though the Italy subtypes were found in other U.S. states, only 88 out of the 1478 sequences from New York in the data set had the same ISM as the two dominant subtypes in Italy (see S6 File). This suggests that the outbreak in New York may not be linked to travel exposure directly from Italy, but rather from another location in Europe, with the important caveat that some potential subtypes may not have been detected there (due to relatively low number of sequences available from Italy). Indeed, the dominant subtype in New York (TCCGCCAGTGG) was detected in 86 sequence from Iceland and only one of them linked to travel exposure in Italy. However, 30 out of 86 cases linked to exposure in Austria, 6 linked to UK, 2 linked to Denmark, and 1 linked to Germany. This further suggests that it was unlikely that the incidence of the TCCGCCAGTGG subtype in New York is connected to Italy but rather than elsewhere in Europe, but limited sequenc [file pcbi.1008269.s002.pdf]

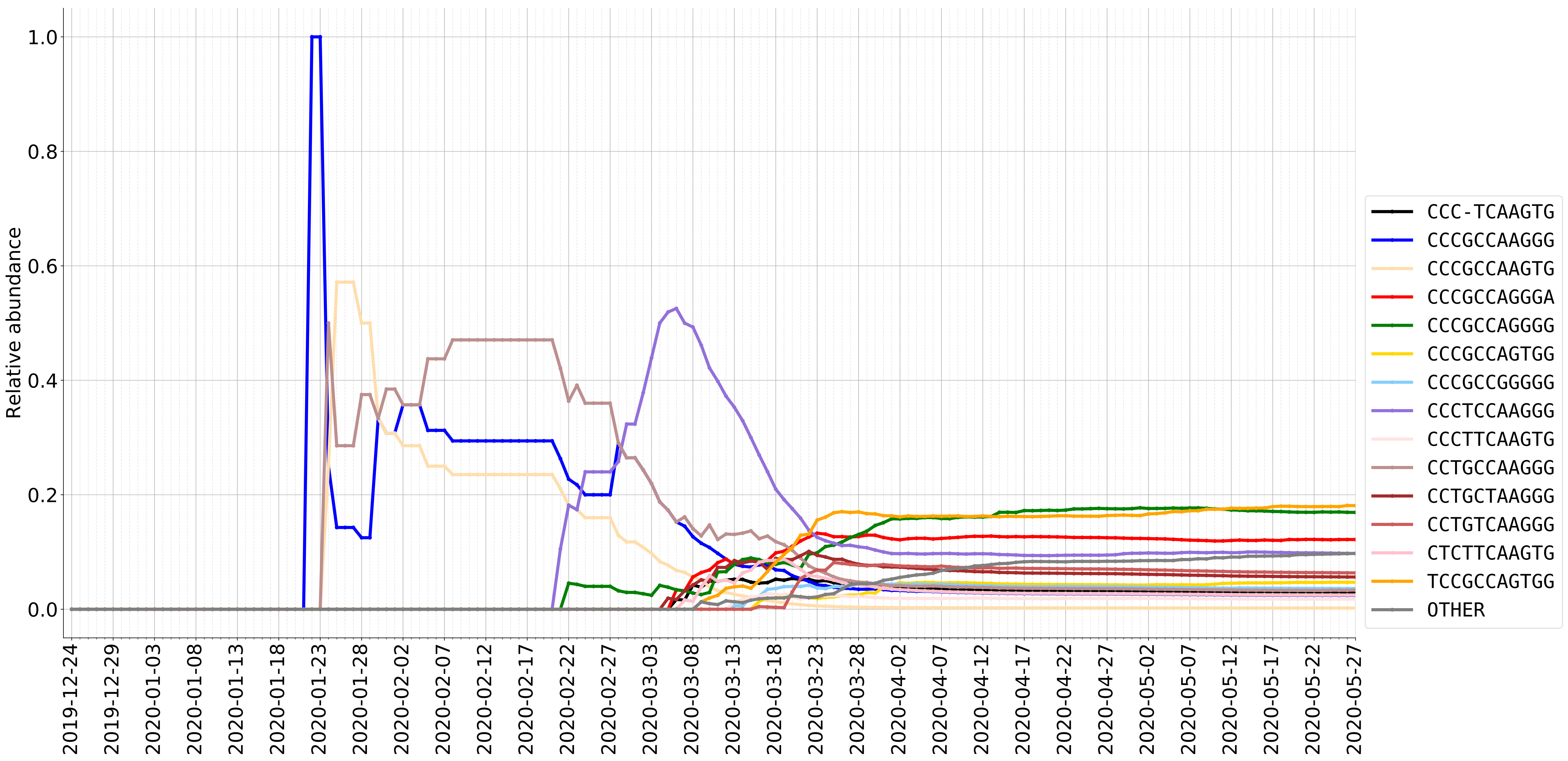

Supplement: S3 Fig — Australia shows growing subtype diversity as its cases increase over time. Initially, Australia’s sequences were dominated by two subtypes that were also substantially abundant in Mainland China (CCCGCCAAGGG and CCTGCCAAGGG). Later, another subtype (CCCTCCAAGGG) starts to emerge. This subtype was less relatively abundant in Mainland China but more highly abundant in sequences from Hong Kong and Singapore (see Fig 5). Then, starting with sequences obtained on February 27, 2020, and subsequently, more subtypes are seen to emerge in Australia that were not found in other Asian countries but were found in Europe. This pattern suggests a hypothesis that Australia may have had multiple independent viral transmissions from Mainland China—or, as noted in the previous discussion, potentially through transmissions from Iran—followed by potentially independent importation of the virus from Europe and North America. (PDF) [file pcbi.1008269.s003.pdf]

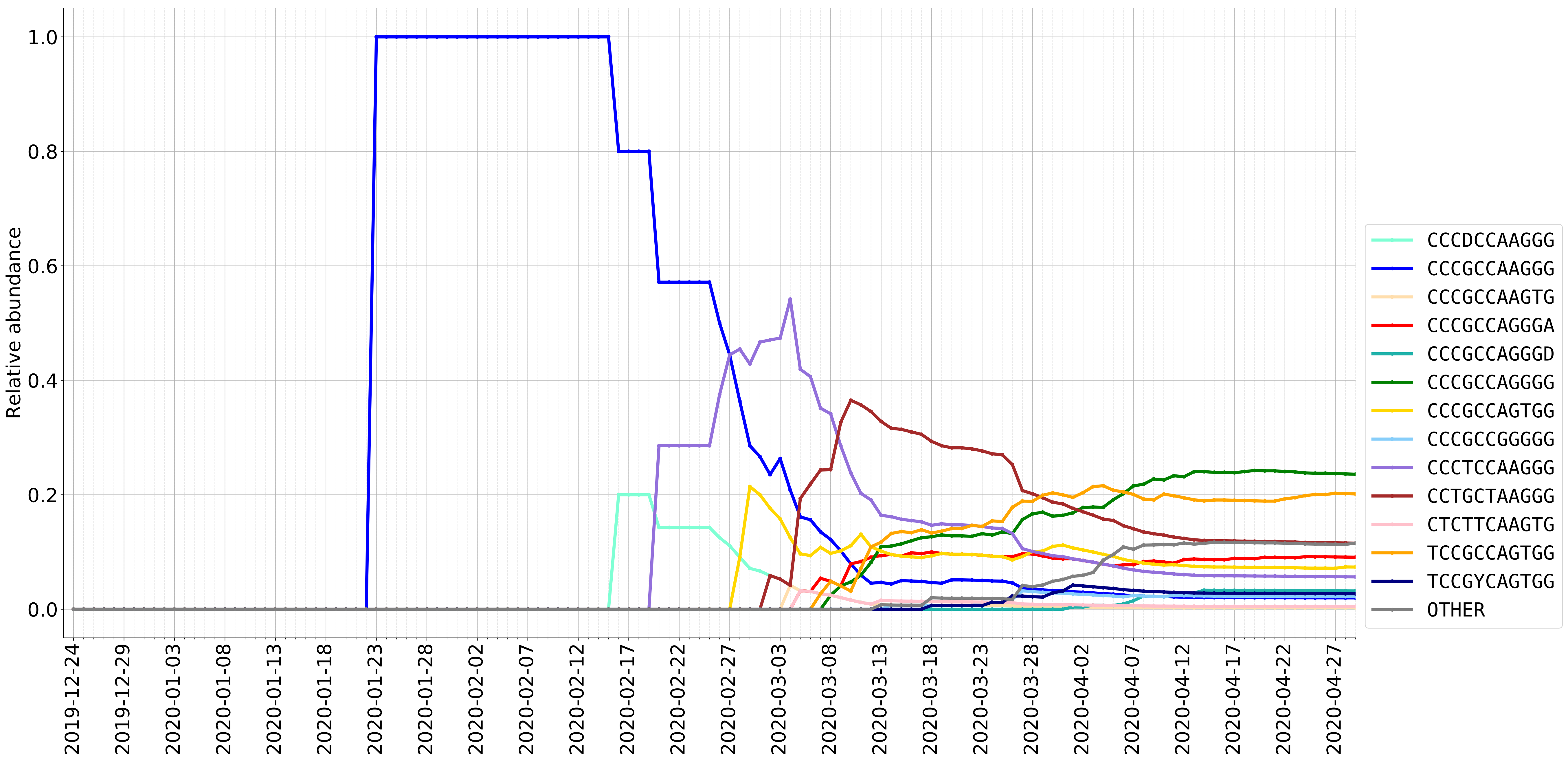

Supplement: S4 Fig — This figure shows that the earliest viral sequences in Canada included mostly subtypes found in Mainland China, with the same pattern in which there was a second, later subtype in common with Mainland China, which was also found in travel exposure from Iran (CCCTCCAAGGG). And, like in Australia, in Canada these few initial viral sequences were followed by a diversification of subtypes that including many in common in Europe and the United States. In sum, Australia and Canada show patterns that might be expected for smaller populations in countries with diverse and extensive travel connections. (PDF) [file pcbi.1008269.s004.pdf]

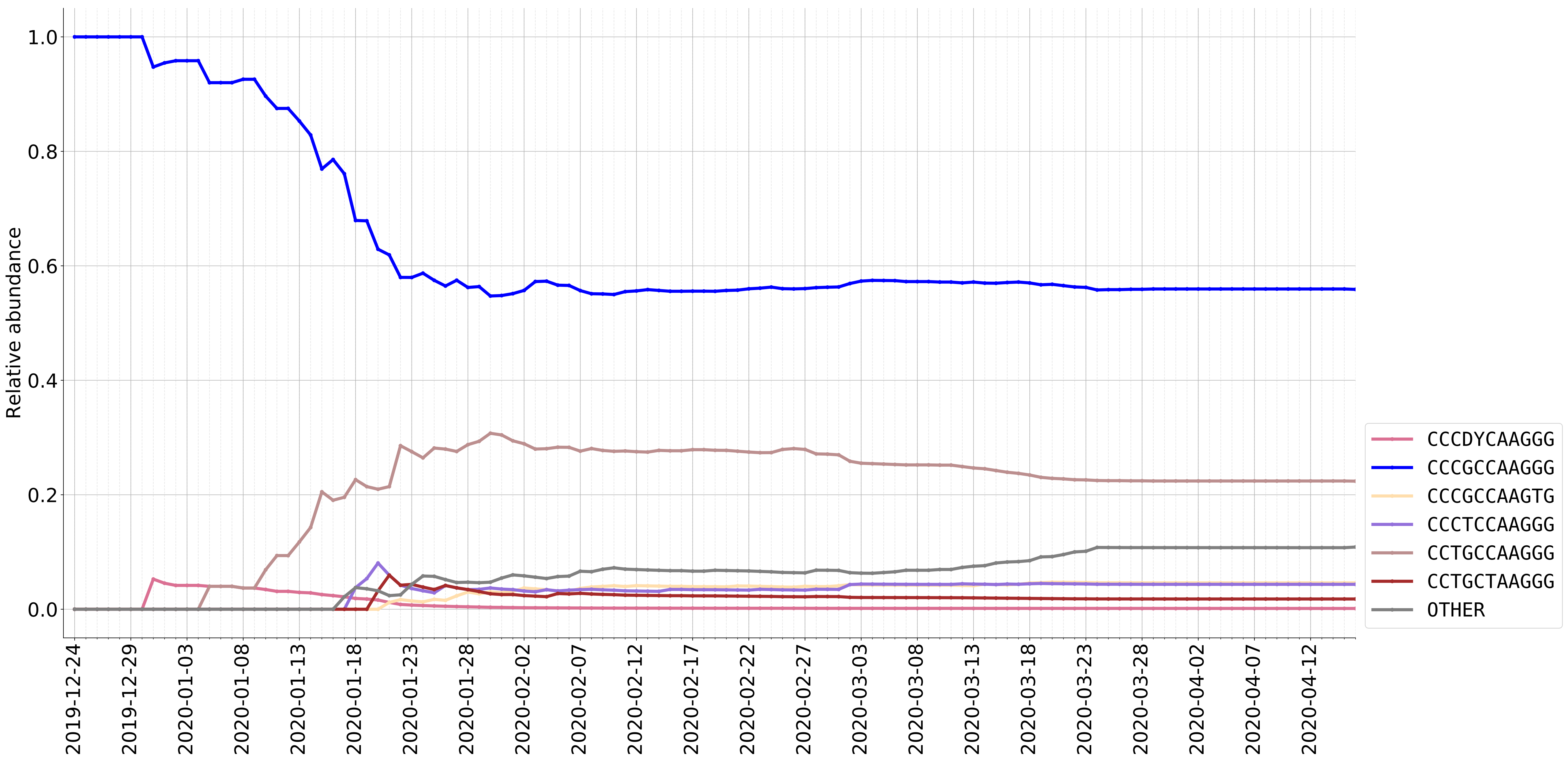

Supplement: S5 Fig — This figure reflects Mainland China’s containment of SARS-CoV-2, as seen in the initial growth in viral genetic diversity, followed by a flattening as fewer new cases were found (and correspondingly fewer new viral samples were sequenced). (PDF) [file pcbi.1008269.s005.pdf]

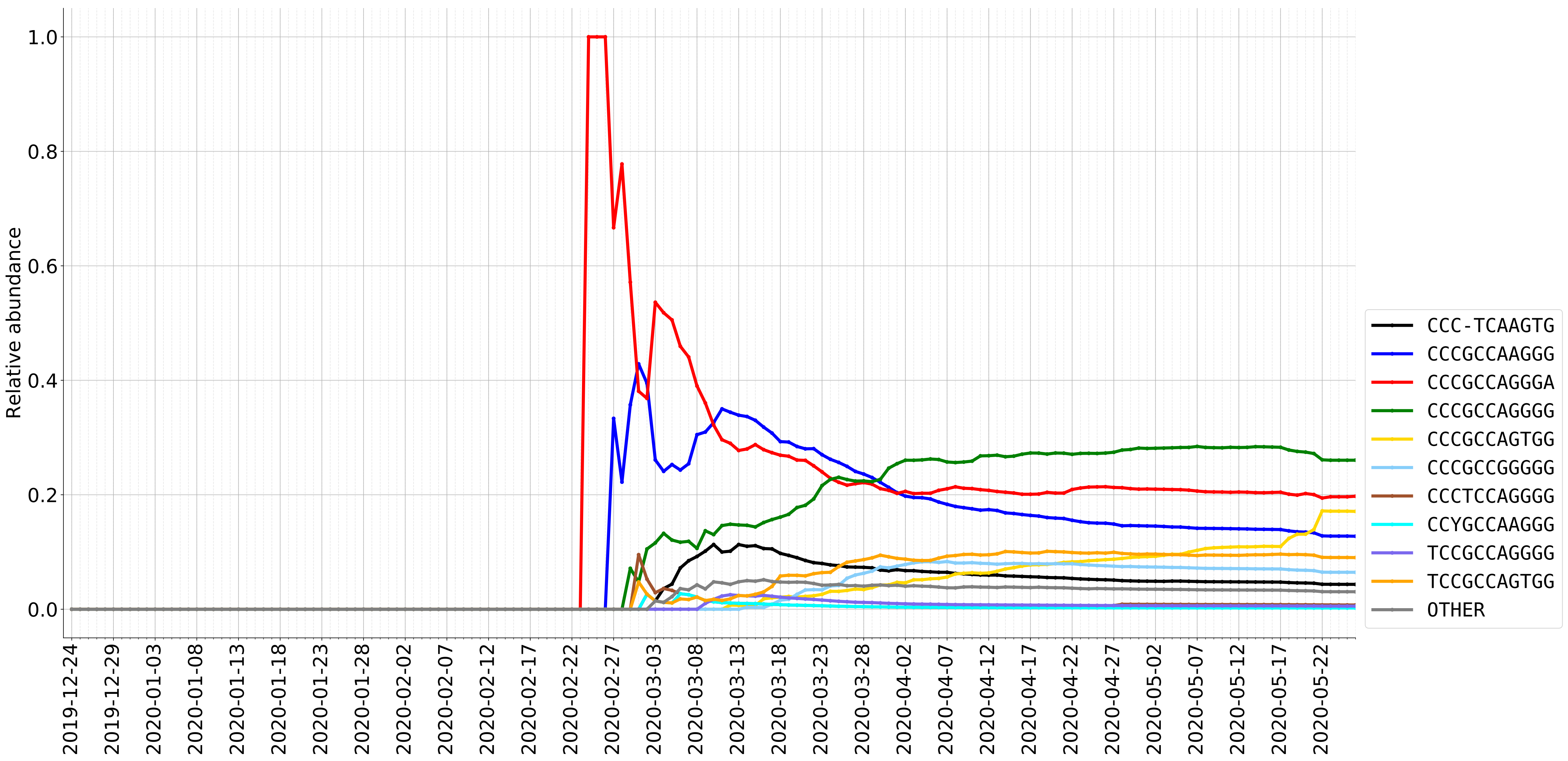

Supplement: S6 Fig — (PDF) [file pcbi.1008269.s006.pdf]

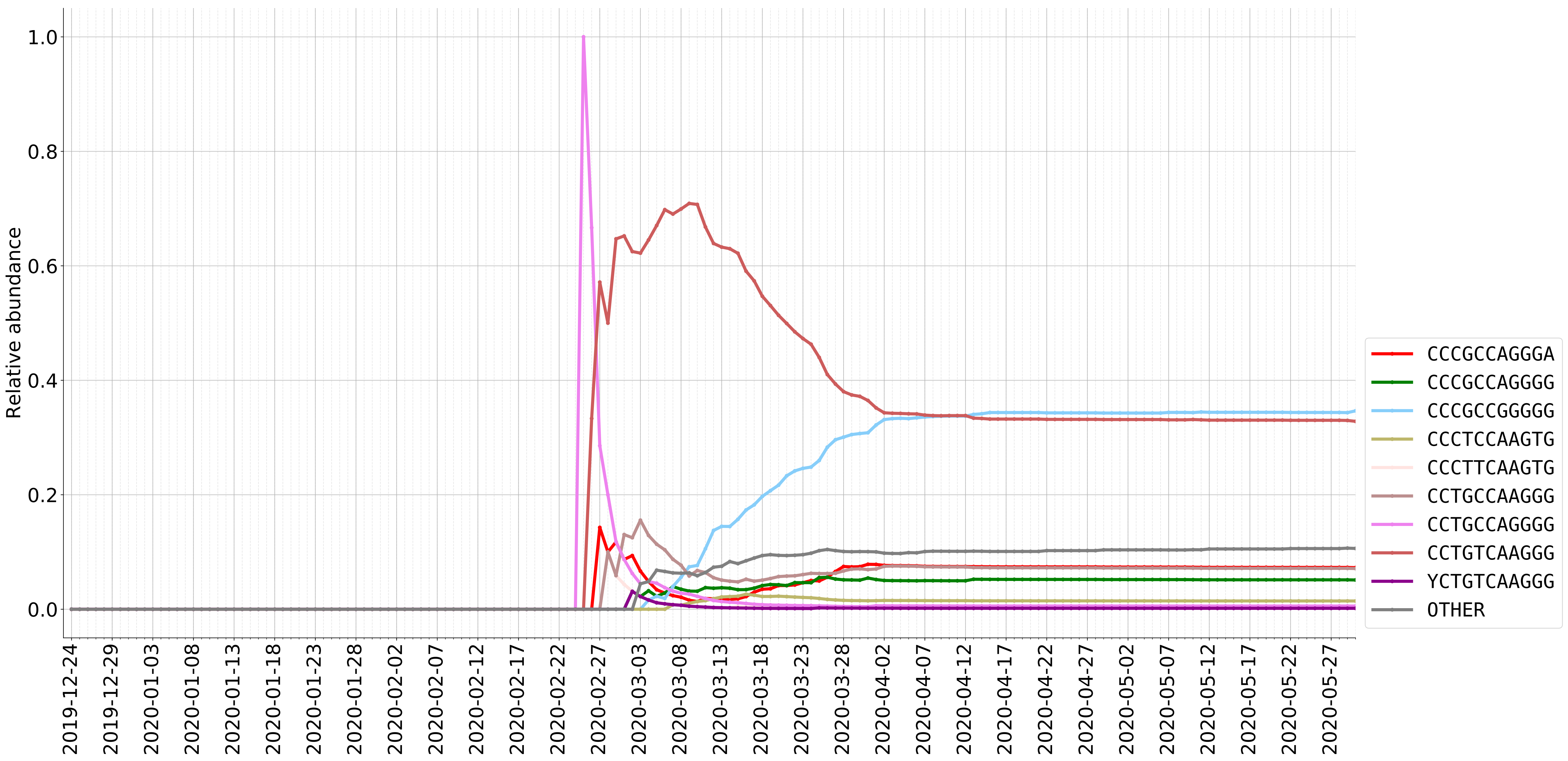

Supplement: S7 Fig — This figure shows, in Spain, the CCCGCCAAGGG subtype was also found in an early sequence data but not thereafter. And, in Spain, a unique subtype has emerged that is not found in abundance in any other country. (PDF) [file pcbi.1008269.s007.pdf]

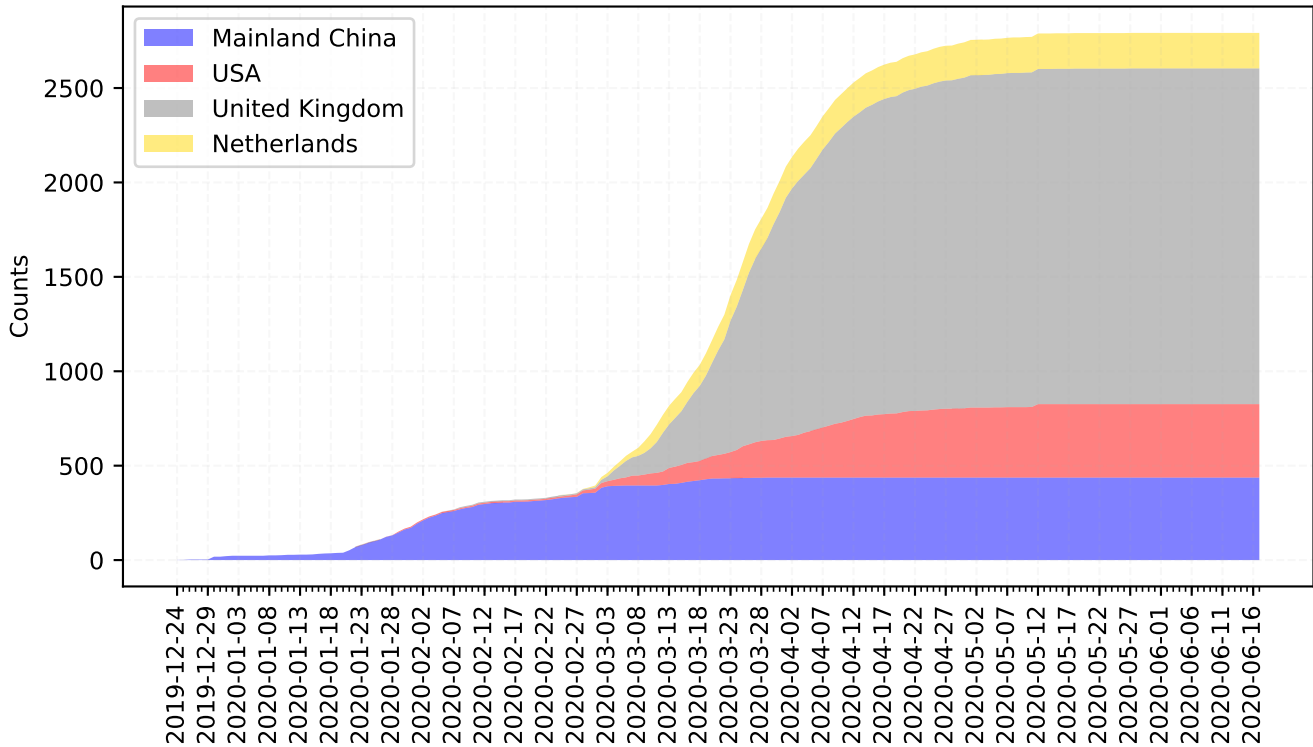

Supplement: S8 Fig — This figure shows that the reference genome subtype began to grow in abundance in Mainland China, before leveling off, and then being detected in the United States and Europe, and subsequently leveling off in those countries as well. In the case of Mainland China, that could be due to the substantial reduction in reported numbers of new infections and thus additional sequences being sampled. However, the other countries have continuing increases in reported infection as of the date of the data set, as well as substantially increasing numbers of sequences being sampled—making it less likely that the reference subtype (CCCGCCAAGGG) is simply being missed. In those cases, it appears from Figs 8 and 9, and S6 Fig that in later times, other subtypes have emerged over time and are becoming increasingly abundant. One potential explanation is that the SARS-CoV-2 is an RNA virus and thus highly susceptible to mutation as transmissions occur [69]. Therefore, as transmissions have continued, the ISM associated with the reference sequence has been replaced by different ISMs due to these mutations. Another plausible explanation for such leveling off in a region is that the leveling off in relative abundance of the subtype represents containment of that subtype’s transmission while other subtypes continue to expand in that country or region. The latter could plausibly explain the pattern observed in the United States, where earlier subtypes connected to Asia did not increase in abundance while a putative endogenous subtype, as well as the dominant New York subtype, have significantly increased in abundance (see Fig 8 and accompanying discussion above). Further investigation and modeling of subtype distributions, as well as additional data, will be necessary to help resolve these questions. (PDF) [file pcbi.1008269.s008.pdf]
