# Supplementary material for: Genetic grouping of SARS-CoV-2 coronavirus sequences using informative subtype markers for pandemic spread visualization
Source: PLoS Comput Biol. 2020 Sep 17;16(9):e1008269. doi: 10.1371/journal.pcbi.1008269 (PMC7523987; doi:10.1371/journal.pcbi.1008269)
Supplement: S4 File — We can see from the figures that major genetic patterns are preserved in compressed ISM system. (PDF) [file pcbi.1008269.s012.pdf]

Visualizations of original ISMs

Zhengqiao Zhao[1*], Bahrad A. Sokhansanj [1†], Charvi Malhotra [2], Kitty Zheng [2], Gail L. Rosen[1+],

1 Ecological and Evolutionary Signal-Processing and Informatics Laboratory, Department of Electrical and Computer Engineering, College of Engineering, Drexel University, Philadelphia, PA, USA
2 College of Medicine, Drexel University, Philadelphia, PA, USA

*** zz374@drexel.edu**
† bahrad@molhealtheng.com
+ glr26@drexel.edu

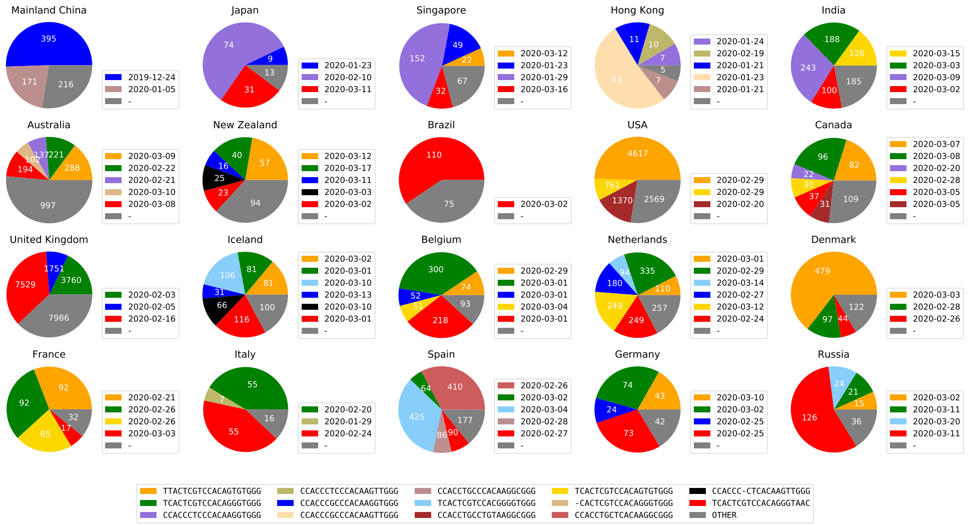

Figure A Major subtypes in countries/regions with the most sequences (indicating date subtype was first sequenced in that country/region). Subtypes with less than 5% abundance are plotted as “OTHER”. The raw counts for all ISMs in each country/region, as well as the date each ISM was first found in a sequence in that country/region

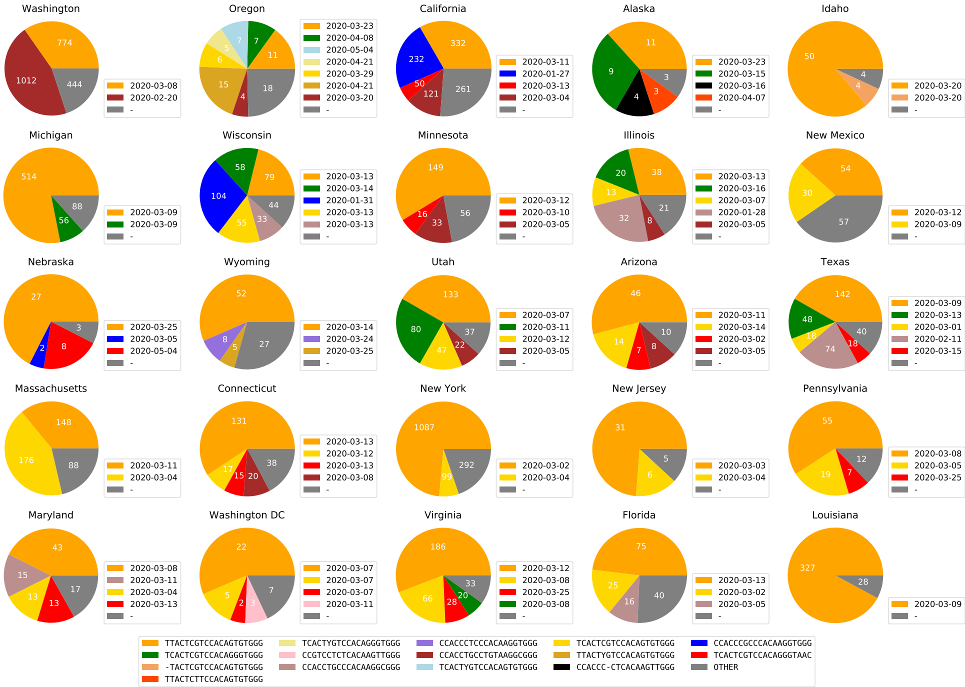

Figure B Viral subtype distribution in the United States, showing the 25 states with the most sequence submissions. Subtypes with less than 5% abundance are plotted as Other. The raw counts for all ISMs in each state, as well as the date each ISM was first found in a sequence in that state

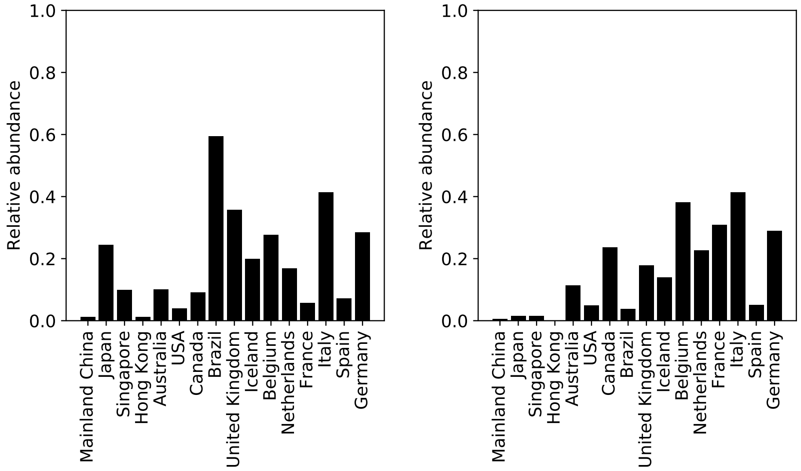

Figure C Relative abundance in other countries of the second-most abundant subtype in Italy, `TCACTCGTCCACAGGGTAAC` (left) and most abundant subtype in Italy `TCACTCGTCCACAGGGTGGG` (right).

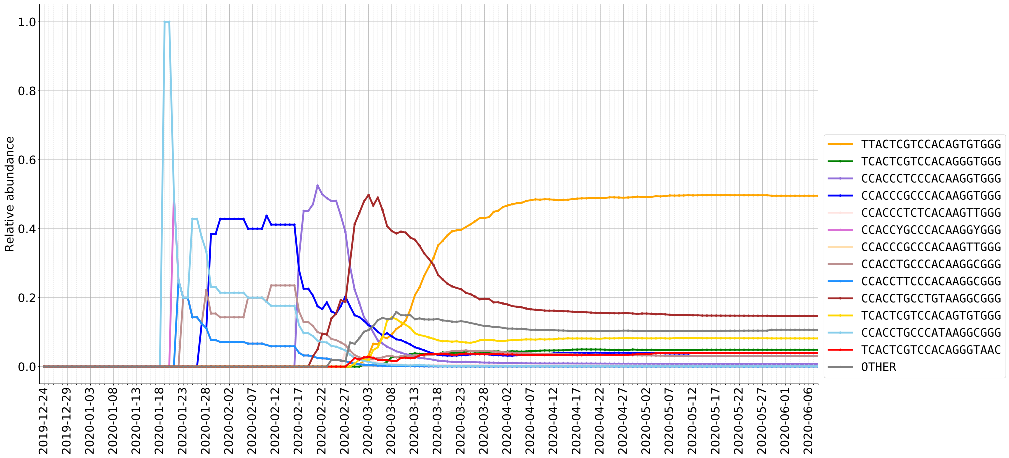

Figure D Relative abundance (%) of ISMs in DNA sequences from USA as sampled over time.

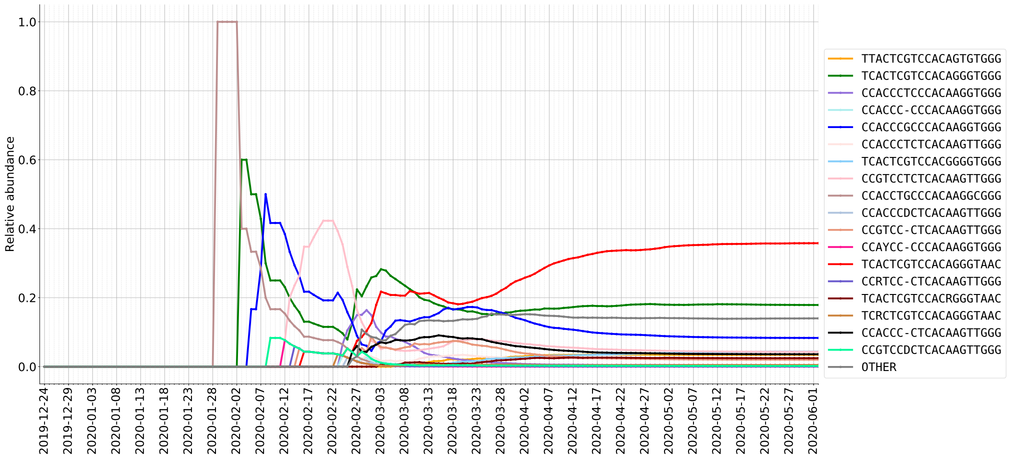

Figure E Relative abundance (%) of ISMs in DNA sequences from USA as sampled over time.

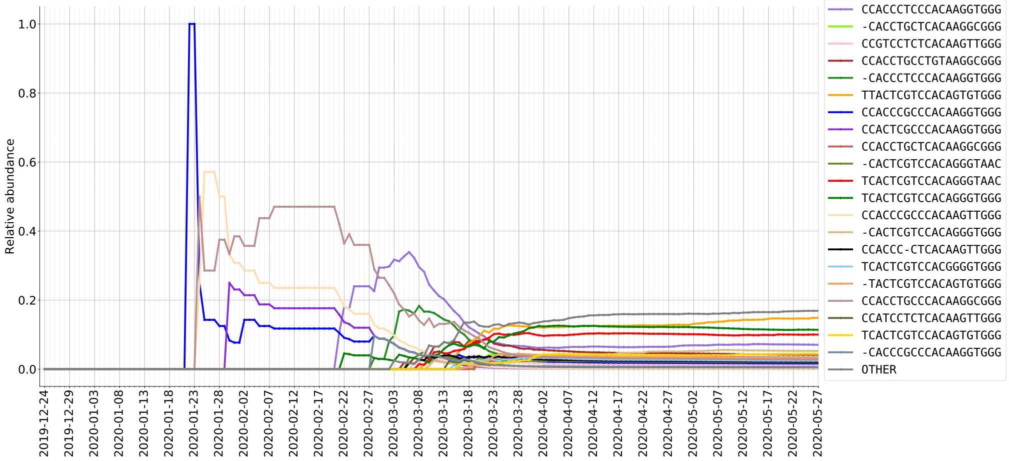

Figure F Relative abundance (%) of ISMs in DNA sequences from Australia as sampled over time.

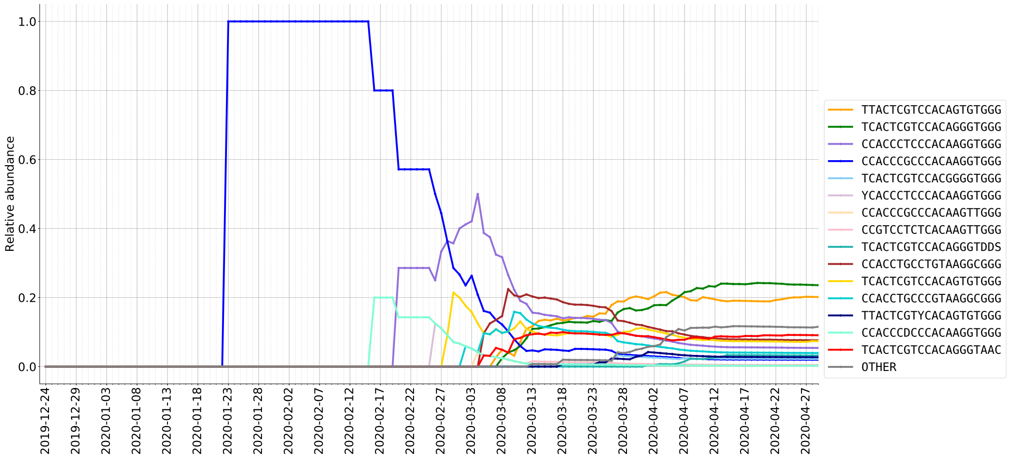

Figure G Relative abundance (%) of ISMs in DNA sequences from Canada as sampled over time.

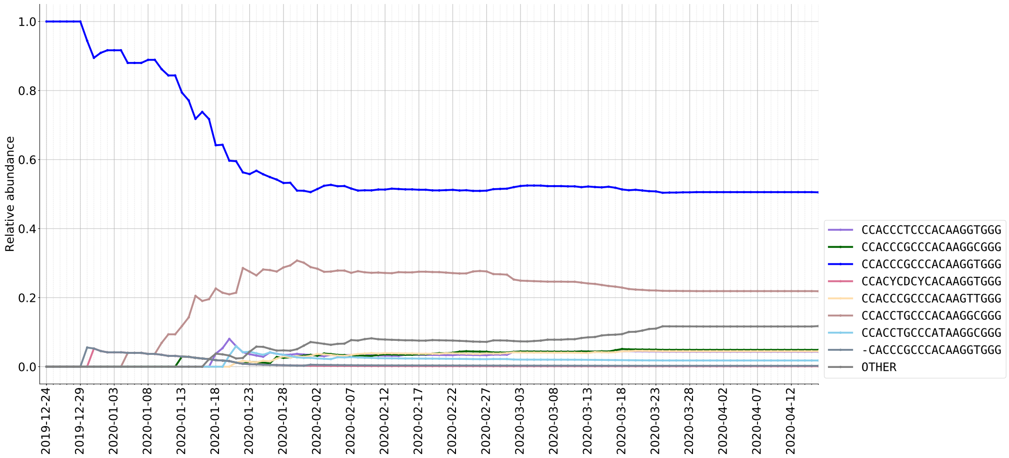

Figure H Relative abundance (%) of ISMs in DNA sequences from Mainland China as sampled over time.

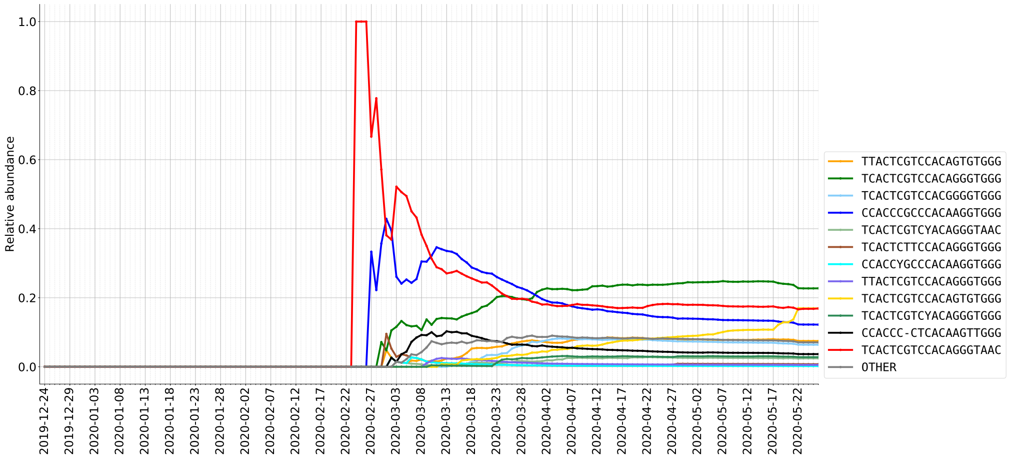

Figure I Relative abundance (%) of ISMs in DNA sequences from Netherlands as sampled over time.

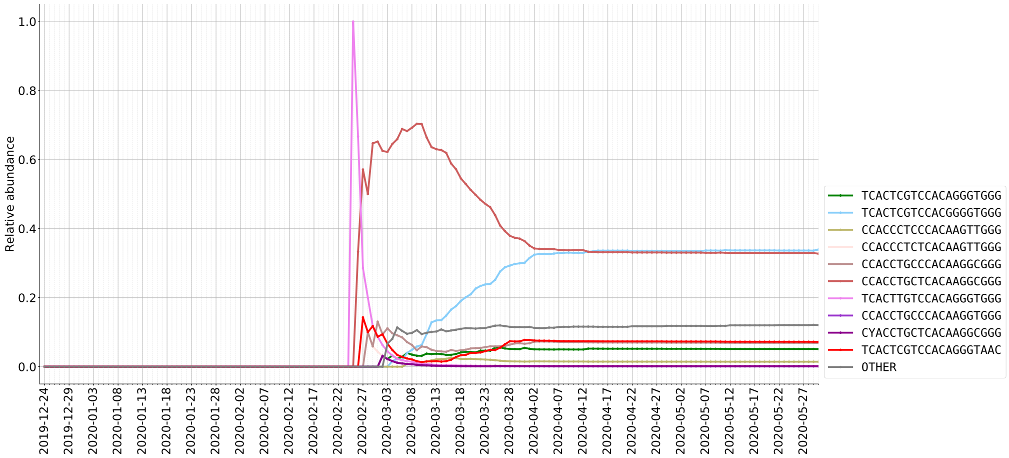

Figure G Relative abundance (%) of ISMs in DNA sequences from Spain as sampled over time.
